# A Plant Endophytic Bacterium, *Burkholderia seminalis* Strain 869T2, Promotes Plant Growth in *Arabidopsis*, Pak Choi, Chinese Amaranth, Lettuces, and Other Vegetables

**DOI:** 10.3390/microorganisms9081703

**Published:** 2021-08-10

**Authors:** Hau-Hsuan Hwang, Pei-Ru Chien, Fan-Chen Huang, Shih-Hsun Hung, Chih-Horng Kuo, Wen-Ling Deng, En-Pei Isabel Chiang, Chieh-Chen Huang

**Affiliations:** 1 Department of Life Sciences, National Chung Hsing University, Taichung 402, Taiwan; so33183.sc@gmail.com (P.-R.C.); seaworld024@hotmail.com (F.-C.H.); walter030170@gmail.com (S.-H.H.); 2 Innovation and Development Center of Sustainable Agriculture, National Chung Hsing University, Taichung 402, Taiwan; chiangisabel@nchu.edu.tw; 3 Institute of Plant and Microbial Biology, Academia Sinica, Taipei 115, Taiwan; chk@gate.sinica.edu.tw; 4 Department of Plant Pathology, National Chung Hsing University, Taichung 402, Taiwan; wdeng@nchu.edu.tw; 5 Department of Food Science and Biotechnology, National Chung Hsing University, Taichung 402, Taiwan

**Keywords:** *Burkholderia*, plant endophytic bacteria, plant-growth-promoting rhizobacteria

## Abstract

Plant endophytic bacteria live inside host plants, can be isolated from surface-sterilized plant tissues, and are non-pathogenic. These bacteria can assist host plants in obtaining more nutrients and can improve plant growth via multiple mechanisms. Certain Gram-negative *Burkholderia* species, including rhizobacteria, bioremediators, and biocontrol strains, have been recognized for their plant-growth-promoting abilities, while other isolates have been identified as opportunistic plant or human pathogens. In this study, we observed the auxin production, siderophore synthesis, and phosphate solubilization abilities of *B. seminalis* strain 869T2. Our results demonstrated that strain 869T2 promoted growth in *Arabidopsis*, ching chiang pak choi, pak choi, loose-leaf lettuce, romaine lettuce, red leaf lettuce, and Chinese amaranth. Leafy vegetables inoculated with strain 869T2 were larger, heavier, and had more and larger leaves and longer and heavier roots than mock-inoculated plants. Furthermore, inoculations of strain 869T2 into hot pepper caused increased flower and fruit production, and a higher percentage of fruits turned red. Inoculation of strain 869T2 into okra plants resulted in earlier flowering and increased fruit weight. In conclusion, the plant endophytic bacterium *Burkholderia seminalis* 869T2 exerted positive effects on growth and production in several plant species.

## 1. Introduction

Under global warming and climate change, cultivated plants are encountering increased biotic and abiotic stresses, which lead to reductions of plant growth and reproduction and consequently economic losses. The use of plant endophytic bacteria to promote plant growth and increase tolerance of environmental stresses has provided an alternative to standard agricultural practices that has fewer safety concerns. Endophytic bacteria can be defined as non-pathogenic bacteria that colonize the interior of host plants and can be isolated from surface-sterilized plant tissues [1]. These bacteria can obtain a constant nutrient supply from host plants by living inside the plants and having close contact with plant cells. The endophytic bacteria colonization process is usually initiated at wounds and cracks in the roots by a rhizospheric population of the bacteria in the soil [2]. After entering the plant roots, endophytic bacteria can systemically colonize the aboveground parts of plants, including stems and leaves [1].

A wide diversity of endophytic bacteria has been discovered in several plant species. Endophytic bacteria communities include five main phyla. Proteobacteria is the most dominant phylum isolated from host plants, which includes α-, β-, and γ-Proteobacteria. Actinobacteria, Planctomycetes, Verrucomicrobia, and Acidobacteria are also commonly identified [2,3]. The most frequently isolated bacteria genera are *Bacillus*, *Burkholderia*, *Microbacterium*, *Micrococcus*, *Pantoea*, *Pseudomonas*, and *Stenotrophomonas*, with the two major genera being *Bacillus* and *Pseudomonas* [4]. Several factors affect the composition of endophytic bacteria populations, including plant growth conditions, plant age, types of analyzed plant tissues, soil contents, and other environmental factors [5].

Endophytic bacteria can have several beneficial effects on host plants, such as promotion of plant growth and yield [1], increased resistance to plant pathogens [6], enhanced tolerance to abiotic stresses [7], elimination of soil pollutants through the facilitation of phytoremediation [8], and production of various metabolites with potential applications in agriculture, medicine, and industry [9]. Some endophytic bacteria help host plants acquire increased amounts of limited resources from the environment. This can include enhancing the uptake of nitrogen, phosphorous, or iron by expressing nitrogenase, solubilizing precipitated phosphates, or producing iron-chelating agents (siderophores) in bacteria, respectively [10,11,12]. Some endophytic plant-growth-promoting bacteria can increase host plants’ metabolism and nutrient accumulation by providing or regulating various plant hormones, including auxin, cytokinin, gibberellins, or ethylene [13,14,15,16]. Auxin and ethylene are the two major hormones that affect plant growth and development and that are involved in plant-endophytic bacteria interactions [1,2]. In addition to these four hormones, several endophytes can utilize signaling pathways mediated by salicylic acid, jasmonic acid, and ethylene to initiate induced systemic resistance (ISR) and protect host plants from phytopathogen infection [3,17]. A number of endophytic bacteria can also produce various antibiotics, toxins, hydrolytic enzymes, and antimicrobial volatile organic compounds to limit pathogen infection [9].

We previously isolated a plant endophytic bacterium, *Burkholderia* sp. strain 869T2, from surface-sterilized root tissues of vetiver grass (*Chrysopogon zizanioides*) [18]. Strain 869T2 can also live within banana (Cavendish cv. Pei-Chiao) plants, in which it promoted growth and reduced *Fusarium* wilt disease occurrence [18]. Genomic sequences of the strain 869T2 contain the gene for 1-aminocyclopropane-1-carboxylate (ACC) deaminase, which may modulate host plant ethylene levels. Strain 869T2 also has genes related to the synthesis of pyrrolnitrin, which may function as a broad-spectrum antifungal agent, as well as dioxin-degradation-related genes [19]. Furthermore, strain 869T2 can degrade the toxic dioxin congener 2,3,7,8-tetrachlorinated dibenzo-p-dioxin (TCDD), mainly via its 2-haloacid dehalogenase (2-HAD) [20]. A recent study compared the genome sequences of 31 *Burkholderia* spp. and reclassified *Burkholderia cenocepacia* strain 869T2 as *Burkholderia seminalis* [21]. We also compared the genome sequences of the strain 869T2 with those of five published *B. seminalis* strains: FL-5-4-10-S1-D7, FL-5-5-10-S1-D0, Bp9022, Bp8988, and TC3.4.2R3 [22,23]. The strain 869T2 shared 93–95% of its genome with the other five *B. seminalis* strains. Furthermore, strain 869T2 lacked several genetic loci that are important for human virulence. Based on the results of our analysis of the core genome phylogeny (1,057 genes) and whole-genome average nucleotide identity (ANI), strain 869T2 was classified as *B. seminalis*.

*B. seminalis* is a member of the *Burkholderia cepacia* complex (Bcc), which is a group of Gram-negative, aerobic, non-sporulating, rod-shaped bacteria [23]. Bcc consists of opportunistic human pathogens that exist in patients suffering from cystic fibrosis (CF) as well as pathogens of many vegetables and fruits, such as onion and banana [24,25,26]. Contrary to the pathogenic traits that led to their original discovery, some Bcc bacteria have ecologically beneficial interactions with host plants [27]. The plant endophytic bacterium *B. seminalis* strain TC3.4.2R3, isolated from sugarcane, can serve as a biocontrol agent to reduce infections with *Fusarium oxysporum* and the cacao pathogens *Moniliophthora perniciosa* (fungus), *Phytophthora citrophtora, P. capsici,* and *P. palmivora* (oomycete) as well as orchid necrosis caused by *Burkholderia gladioli* through the production of pyochelin, a rhamnolipid, and other unidentified diffusible metabolites [28,29,30]. Another strain of *Burkholderia seminalis,* strain R456 isolated from rice rhizosphere soils, decreased the occurrence of rice sheath blight (ShB) disease caused by *Rhizoctonia solani* [31,32]. Furthermore, *Burkholderia seminalis* strain ASB21 was found to be able to produce the plant hormone auxin, promote rice seedling growth, and reduce aluminum toxicity symptoms in host plants [33]. Similarly, a *Burkholderia seminalis* strain isolated from Bangalore, India can produce indole acetic acid (IAA) and enhance tomato seedling growth [34].

Although it is known that *Burkholderia seminalis* belongs to the plant-growth-promoting rhizobacteria (PGPR), only limited strains and their promoting abilities are well characterized. In this study, we examined the amounts of IAA produced by *B. seminalis* strain 869T2 in various growth conditions, detected the strain’s siderophore synthesis and phosphate solubilization abilities, and demonstrated its growth-promoting abilities in several leafy vegetables, including pak choi, lettuce, and amaranth.

## 2. Materials and Methods

### 2.1. Bacterial Strains and Culture Conditions

*B. seminalis* strain 869T2 was isolated from the roots of surface-sterilized vetiver grass (*Chrysopogon zizanioides*) [18]. Strain 869T2 was grown in Luria Broth (LB) media (1.0% tryptone, 0.5% yeast extract, 0.5% NaCl, pH 7.5) or on LB agar with appropriate antibiotics (ampicillin 100 μg mL^−1^) at 30 °C. *Escherichia coli* strain DH10B was cultured in LB media at 37 °C and was used as the negative control in the siderophore synthesis and phosphate solubilization assays. For growth tests of strain 869T2 in various conditions, bacteria were grown on LB media at 20 °C, 25 °C, 30 °C, 37 °C, or 45 °C; on LB media with pH 4.0, 6.0, 7.5, or 9.0; or on M9 salt media (pH 7.5) with 2% glucose, fructose, or sucrose at 30 °C. Bacterial cells were measured at OD_600_ to determine their biomass and were grown for 48 h. Three biological replicates were used for each growth measurement.

### 2.2. Colorimetric Assay for Indole Acetic Acid (IAA) Determination

Indole acetic acid (IAA) production was determined as described previously [35], with minor modifications. Bacterial cultures were grown on LB media containing 100 μg mL^−1^ of tryptophan with different pH levels (pH 4.0, 6.0, 7.5, or 9.0) and appropriate antibiotic for 48 h at selected temperatures (20 °C, 25 °C, 30 °C, 37 °C, or 45 °C). Bacteria were also cultured on M9 salt media (pH 7.5) at 30 °C for 48 h with 100 μg mL^−1^ of tryptophan and 2% of different kinds of sugar: glucose, fructose, or sucrose. Fully grown bacteria cultures were then centrifuged at 5000 rpm for 10 min, and the supernatant was passed through a syringe filter with a pore size of 0.2 µm (Sartorius, Goettingen, Germany) to remove bacteria. The 500 μL of supernatant was mixed with 1 mL of the Salkowski reagent (50 mL of 36% of H_2_SO_4_ and 1 mL 0.5 M FeCl_3_ solution) and incubated at room temperature for 25 min. Finally, the concentrations of IAA in the supernatants were determined by comparison of the absorbance measured at 530 nm (OD_530_) with a standard curve of 0–100 µg mL^−1^ IAA.

### 2.3. Detection of Siderophore Synthesis

Siderophore synthesis was determined as described by Schwyn and Neilands (1987) [36] with minor modification. Strain 869T2 was grown on King’s medium B (KB medium) plates at 30 °C overnight, and then chrome azurol S (CAS) agarose solution was applied over the agar plates. After incubation for 2 h at room temperature, development of the initial blue color to a yellow or orange color was indicative of siderophore production.

### 2.4. Detection of Phosphate Solubilization

The phosphate solubilization activity of strain 869T2 was determined using Pikovskaya’s agar medium with 0.5% tricalcium phosphate (Ca_3_(PO_4_)_2_) as the inorganic phosphate source. The bacteria culture was spot-inoculated in plates and incubated at 28 °C for 72 h. The formation of a clear zone around the bacteria colony indicated phosphate solubilization [37].

### 2.5. Inoculations and Reisolation Assays of the B. seminalis Strain 869T2 with Different Plant Species

Different plant species underwent bacteria inoculation assays according to the protocols of Ho et al. [18]. Seeds or seedlings of ching chiang pak choi (*Brassica chinensis*), pak choi (*Brassica rapa* L. R. Chinensis Group), loose-leaf lettuce (*Lactuca sativa* L.), romaine lettuce (*Lactuca sativa* L. var. *romana*), red leaf lettuce (*Lactuca sativa* L. var. *crispa*), Chinese amaranth (*Amaranthus tricolor*), hot pepper (*Capsicum annuum*), and okra (*Abelmoschus esculentus*) were obtained from the Known-You seed company (Kaohsiung, Taiwan), and grown in soils at 24 to 26 °C (16 h light/8 h dark). *Arabidopsis* seeds (ecotypes Columbia, BL-1, UE-1, and Dijon-G) were also germinated and grown in soils identical to the other types of plant seedlings. The 4- to 6-leaf seedlings grown in pots were then inoculated with *B. seminalis* strain 869T2.

The *B. seminalis* 869T2 cells were grown on LB media (pH 7.5) with antibiotics at 30 °C to an approximate OD_600_ value of 0.6–0.8. Bacterial cultures were then collected and adjusted to approximately 10^8^ cfu mL^−1^, as determined by plate counting. Five milliliters of bacterial culture were added to the potting soils of each plant seedling. After inoculations, plant seedlings were grown in soil in either greenhouses or fields for 14 to 80 days. At least 20 individual seedlings of each kind of plant were inoculated with strain 869T2. Three independent bacteria inoculation assays were performed for every kind of plant, and more than 60 individual plants were examined in the bacteria inoculation assays.

In order to confirm the endophytic colonization of the plants by strain 869T2, reisolation of bacteria from the inoculated plants was performed as described by Ho et al. [18] with minor modifications. Seven days after inoculation, the inoculated plant tissues were surface-sterilized with 70% ethanol for 1 min followed by 10% commercial chlorine bleach and a 0.1% Tween 20 solution for 10 min, then washed three times in sterile distilled water. The sterilized plant tissues were then macerated with sterile distilled water in sterile mortars to obtain an aqueous extract. Suitable amounts of extracts were serially diluted and plated on LB agar with antibiotics to determine the number of viable bacterial cells. Bacteria were identified via sequencing and phylogenetic analysis of the 16S ribosomal RNA (16S rRNA) gene. The 16S rRNA gene was amplified by colony PCR reactions with primers E8F (5′-AGAGTTTGATCATGGCTCAG-3′) and U1510R (5′-CGGTTACCTTGTTACGACTT-3′) [18].

### 2.6. Measurements of Plant Growth Parameters

Various growth parameters of different plant species were measured at selected days, ranging from 14 to 80 days, after inoculation with strain 869T2. The fresh weight, dry weight, and length of leaves and roots as well as the width, number, and surface area of leaves were measured in harvested pak choi, lettuce, and Chinese amaranth as described previously [38,39,40]. The fresh weight, length, number, and color of fruits of hot pepper and okra were recorded following previously described methods [41,42]. The chlorophyll content of the lettuce leaves was measured using a previously published protocol [43]. Chlorophyll was extracted from the leaves with N, N-Dimethylformamide (DMF) for 1 hour in the dark, and chlorophyll a and b concentrations were calculated from the absorbance of the crude extract at 647 and 664 nm. Anthocyanin concentrations were determined using a published acidified methanol method [44]. Hot pepper fruits were first ground with liquid nitrogen. Acidified (1% HCl) methanol was then mixed with the ground materials for 10 min in darkness with shaking. These crude extracts were subsequently mixed with an extraction solvent containing 1:1 chloroform:water (*v*/*v*) to isolate anthocyanins. After centrifugation, the absorbance of the supernatant was read at 530 and 657 nm by the spectrophotometer, and anthocyanin contents were calculated from these values.

### 2.7. Statistical Analysis

The growth measurements were average values from at least three independent bacteria inoculation experiments, each containing at least 20 individual plants. Error bars were calculated by using the Microsoft Excel STDEVP function. The significance test between treatments was based on Duncan tests or pairwise Student *t*-test, with *p* < 0.05 considered statistically significant.

## 3. Results

### 3.1. Effects of Temperature, pH, and Carbon Sources on the Synthesis of IAA by the B. seminalis Strain 869T2

**Figure 1:**
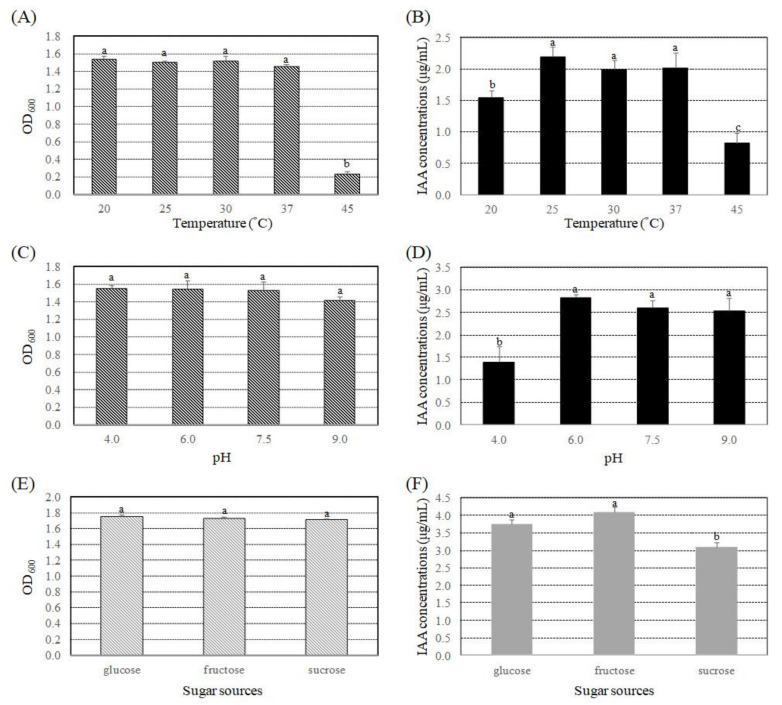
Effects of temperature, pH, and different sugar sources on bacteria growth and IAA production by *B. seminalis* strain 869T2. Strain 869T2 was cultured in LB media (pH 7.5) at 20, 25, 30, 37, or 45 °C for 48 h to determine bacteria growth (OD_600_) (**A**) and IAA production (**B**). Strain 869T2 was also grown in LB media at pH 4.0, 6.0, 7.5, or 9.0 at 30 °C for 48 h to determine bacteria growth (OD_600_) (**C**) and IAA production (**D**). Finally, the tested bacteria were cultivated in M9 salt media (pH 7.5) with 2% glucose, fructose, or sucrose at 30 °C for 48 h to determine bacteria growth (OD_600_) (**E**) and IAA production (**F**). Data are mean ± SE (standard error) from at least three independent bacteria growth experiments. Data were analyzed using Duncan tests and means with different letters were significantly different (*p* < 0.05).

A previous study revealed that *B. seminalis* strain 869T2 produces the plant hormone indole-3-acetic acid (IAA) [18]. Various growth conditions, including temperature, pH, and different carbon sources, can affect the synthesis of IAA by bacteria [45,46,47]. To determine the effect of temperature on IAA production by strain 869T2, bacteria cultures were grown on LB media (pH 7.5) at 20 °C, 25 °C, 30 °C, 37 °C, or 45 °C for 48 h. Strain 869T2 grew well at temperatures of 20 °C to 37 °C, but not at 45 °C (Figure 1A). The results shown in Figure 1B indicate that when strain 869T2 was grown at 25 °C, 30 °C, or 37 °C, the bacterial cultures had the highest IAA yields of 2.0 to 2.2 μg mL^−1^, followed by the temperature of 20 °C, with the lowest IAA yield of 0.8 μg mL^−1^ at 45 °C. These data suggest that too high a temperature may affect both bacteria growth and IAA production.

The effects of pH were also examined by culturing strain 869T2 in LB media at 30 °C over a pH range of 4 to 9. Strain 869T2 was able to grow over this entire pH range (Figure 1C). The results shown in Figure 1D demonstrate that IAA production was at a similar level when bacteria were grown at pH 6 to 9, whereas the IAA amount decreased 44.0% when bacteria were grown at pH 4. Additionally, three different sugars, glucose, fructose, and sucrose, were used in the minimal medium (M9 salt medium, pH 7.5) to examine the effects of different carbon sources on IAA production. Strain 869T2 grew similarly in the M9 salt media with different kinds of sugars (Figure 1E). The results shown in Figure 1F indicate that when strain 869T2 was grown in the media with two kinds of monosaccharide, glucose and fructose, the IAA amounts were higher than for the bacteria grown in the media with sucrose.

We further investigated whether strain 869T2 had other plant-growth-promoting traits, including siderophore production and phosphate solubilization abilities, with agar plate assays. Supplementary Materials Figure S1A shows that the strain 869T2 colonies exposed to CAS agarose turned yellow, indicating the siderophore production ability of strain 869T2. Furthermore, Figure S1B reveals that the formation of halos around the strain 869T2 colonies grown in Pikovskaya’s agar medium with 0.5% tricalcium phosphate suggests that strain 869T2 may have the ability to solubilize phosphate.

### 3.2. Effect of the B. seminalis Strain 869T2 on Growth of Arabidopsis thaliana and Two Vegetables of the Brassica Genus

**Figure 2:**
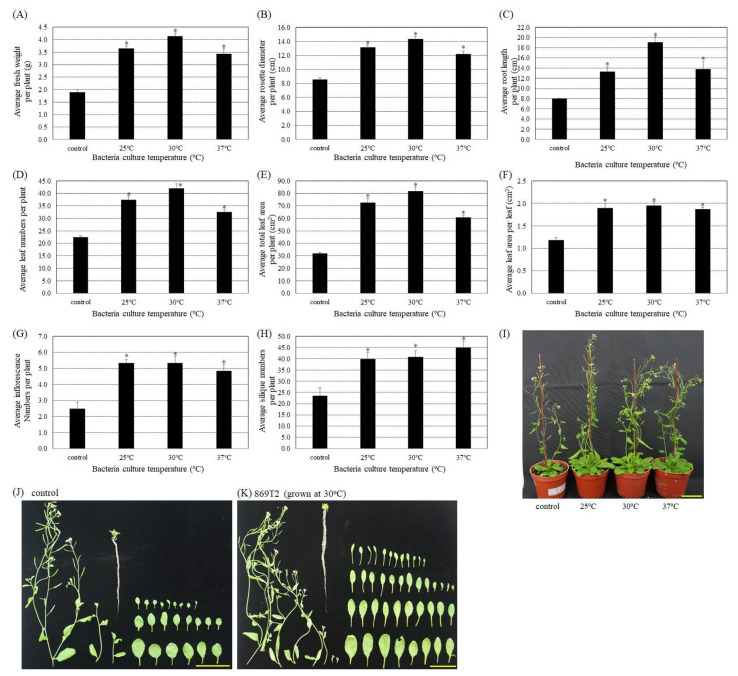
Inoculation of *B. seminalis* strain 869T2 into *Arabidopsis thaliana* (ecotype: Columbia) improved various growth parameters compared with mock-inoculated control plants. Strain 869T2 was grown at 25, 30, or 37 °C and then used to inoculate *Arabidopsis* plants. Two weeks after inoculation, the average values for fresh weight (**A**), rosette diameter (**B**), root length (**C**), leaf numbers per plant (**D**), total leaf area per plant (**E**), leaf area per leaf (**F**), and the numbers of inflorescences (**G**), and siliques (**H**) per plant were determined for the inoculated and control plants. Data are mean ± SE (standard error) from at least three independent bacteria inoculation experiments. More than 20 individual plants were examined for each bacteria inoculation assay. * *p* < 0.05 compared with the control plants by pairwise Student *t*-tests. (**I**), photographs of the mock-inoculated control *Arabidopsis* plant and the plants inoculated with strain 869T2 cultured at 25 °C, 30 °C, or 37 °C. Photographs of total leaves, inflorescences, and roots of the control (**J**) and the 869T2-inoculated plants (**K**). Yellow bar = 5 cm.

A previous study by Ho et al. [18] demonstrated that strain 869T2 promoted plant growth in banana, a monocot. Here, the growth promotion ability of strain 869T2 was tested in three different eudicot plants from the Brassicaceae family, namely *Arabidopsis thaliana*, ching chiang pak choi, and pak choi. Because strain 869T2 produced relatively higher amounts of IAA at 25 °C to 37 °C (Figure 1B), we cultured strain 869T2 at three different temperatures, 25 °C, 30 °C, and 37 °C. Subsequently *Arabidopsis thaliana* ecotype Columbia was inoculated with these strains to determine which strain had the best plant growth promotion ability. We confirmed the endophytic colonization of the *Arabidopsis* plants by strain 869T2 by reisolating the bacteria from surface-sterilized inoculated plant tissues. The identities of the isolated bacteria were determined via sequencing and phylogenetic analysis of the 16S ribosomal RNA (16S rRNA) gene. Subsequently, different plant growth parameters were examined in *Arabidopsis* plants inoculated with strain 869T2 and in mock-inoculated controls. Two weeks after inoculation, the presence of strain 869T2 increased the average fresh weight (Figure 2A), rosette diameter (Figure 2B), root length (Figure 2C), number of leaves (Figure 2D), total leaf area per plant (Figure 2E), leaf area per leaf (Figure 2F), number of inflorescences (Figure 2G), and number of siliques (Figure 2H) of *Arabidopsis* plants more than 1.5- to 2.1-fold compared with mock-inoculated controls. As shown in Figure 2I–K, the overall size and number of leaves of plants inoculated with strain 869T2 were greater than those of control plants, indicating that strain 869T2 promoted *Arabidopsis* plant growth.

Furthermore, when the plants were inoculated with strain 869T2 grown at 30 °C, the average root length (Figure 2C) and average total leaf area per plant (Figure 2E) were slightly higher than for the strains grown at 25 °C and 37 °C. We therefore tested whether strain 869T2 grown at 30 °C could enhance the growth of different *Arabidopsis* ecotypes and other plant species. In addition to *Arabidopsis* ecotype Columbia, three *Arabidopsis* ecotypes that are less susceptible to *Agrobacterium tumefaciens* infection, BL-1, UE-1, and Dijon-G [48,49], were selected to examine the growth promotion ability of strain 869T2. After inoculation with strain 869T2, the average value of the fresh weight (Figure S2A), dry weight (Figure S2B), rosette diameter (Figure S2C), root length (Figure S2D), number of leaves (Figure S2E), total leaf area per plant (Figure S2F), and leaf area per leaf (Figure S2G) of the three additional *Arabidopsis* ecotypes were 1.2- to 2.0-fold higher than control plants. These data further support the hypothesis that the presence of strain 869T2 in different *Arabidopsis* ecotypes has a positive impact on plant growth.

**Figure 3:**
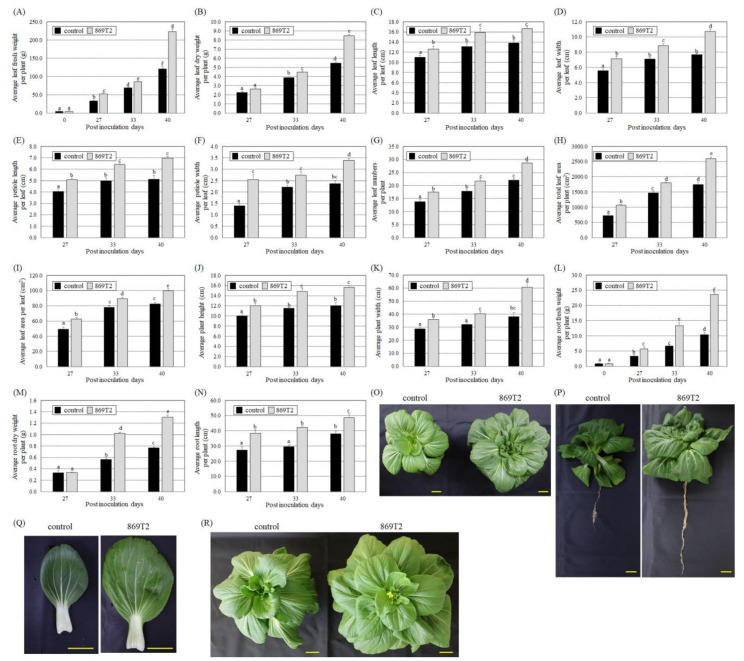
*B. seminalis* strain 869T2 enhanced the growth of ching chiang pak choi (*Brassica chinensis*) plants. At 27, 33, and 40 days after inoculation with strain 869T2, the average values for leaf fresh weight (**A**) and leaf dry weight (**B**) per plant, leaf length (**C**), leaf width (**D**), petiole length (**E**), petiole width (**F**), number of leaves (**G**), total leaf area (**H**) per plant, leaf area per leaf (**I**), plant height (**J**), plant width (**K**), root fresh weight (**L**), root dry weight (**M**), and root length (**N**) were recorded for the control and inoculated plants. Data are mean ± SE (standard error) from at least three independent bacteria inoculation experiments. More than 20 individual plants were examined for each bacteria inoculation assay. Data were analyzed using Duncan tests and means with different letters were significantly different (*p* < 0.05). Panels (**O**–**Q**): top-view (**O**), side-view (**P**), and single leaf (**Q**) photographs of the mock-inoculated control and the 869T2-inoculated plants at 40 days after inoculation. top-view photographs of the control and 869T2-inoculated plants 53 days after inoculation (**R**). Yellow bar = 5 cm.

Seedlings of ching chiang pak choi (*Brassica chinensis*) and pak choi *(Brassica rapa* L. R. Chinensis Group) from the *Brassica* genus were also inoculated with strain 869T2 to examine its effects on plant growth. At 27, 33, and 40 days after inoculation with strain 869T2, the average fresh weight and dry weight of aboveground leaves of ching chiang pak choi were higher than those of the control plants (Figure 3A,B). Furthermore, the average leaf length and width, petiole length and width, number of leaves per plant, total leaf area per plant, and leaf area per leaf were greater in the 869T2-inoculated ching chiang pak choi compared to the control plants (Figure 3C–I). The results shown in Figure 3J,K demonstrate that the average plant height and width of the 869T2-inoculated ching chiang pak choi were also greater compared to the control plants. Similarly, after the ching chiang pak choi was inoculated with strain 869T2, the average values of root fresh weight, dry weight, and length were higher in comparison to control plants (Figure 3L–N). Figure 3O–Q indicate that both the aerial and belowground parts of ching chiang pak choi were larger after inoculation with strain 869T2. Figure 3R also shows that the ching chiang pak choi inoculated with strain 869T2 grew faster and flowered earlier than control plants 53 days after inoculation. Similarly, after inoculation with strain 869T2, the pak choi grew larger, including larger and more numerous leaves, larger aerial parts overall, and longer and heavier roots (Figure S3). These data indicate that inoculation of strain 869T2 in two vegetables from the Brassicaceae family significantly improved their growth.

### 3.3. Inoculations of Lettuce and Chinese Amaranth with the B. seminalis Strain 869T2 Promoted Plant Growth

**Figure 4:**
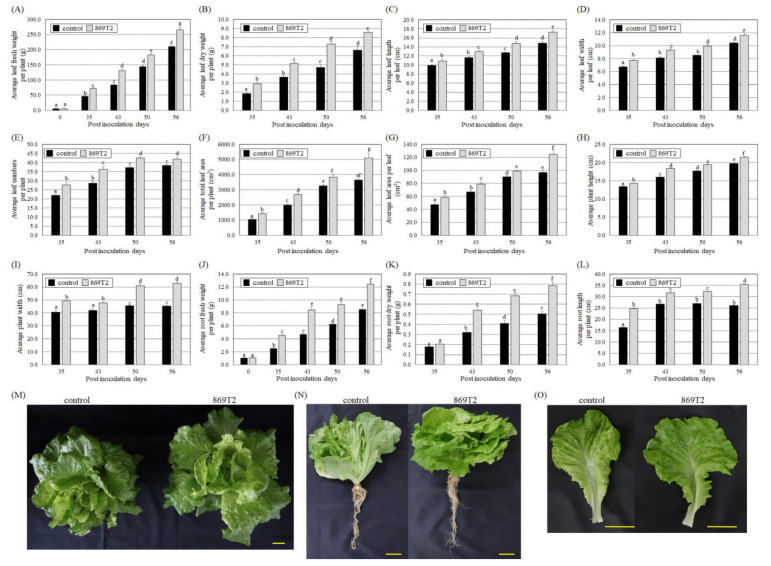
Loose-leaf lettuce (*Lactuca sativa* L.) plants were larger and heavier after inoculation with *B. seminalis* strain 869T2. At 35, 43, 50, and 56 days after inoculation, the average values for leaf fresh weight (**A**), leaf dry weight (**B**), leaf length (**C**), leaf width (**D**), number of leaves (**E**), total leaf area per plant (**F**), leaf area per leaf (**G**), plant height (**H**), plant width (**I**), root fresh weight (**J**), root dry weight (**K**), and root length (**L**) were examined for the control and inoculated plants. Data are mean ± SE (standard error) from at least three independent bacteria inoculation experiments. More than 20 individual plants were examined for each bacteria inoculation assay. Data were analyzed using Duncan tests and means with different letters were significantly different (*p* < 0.05). Panels (**M**–**O**): top-view (**M**), side-view (**N**), and single leaf (**O**) photographs of the control and 869T2-inoculated plants 56 days after inoculation. Yellow bar = 5 cm.

Because *B. seminalis* strain 869T2 successfully colonized *Arabidopsis* and two types of plants from the Brassicaceae family and promoted their growth, we further examined whether strain 869T2 could promote the growth of plants from the Asteraceae and Amaranthaceae families. At 35, 43, 50, and 56 days after inoculation with strain 869T2, the fresh weight of the aerial parts of inoculated loose-leaf lettuce plants increased 12.7- to 46.6-fold compared to the 0-day post-inoculation plants (Figure 4A). By comparison, in the mock-inoculated control plants, the fresh weight increased 8.0- to 36.0-fold over the same period (Figure 4A). Similarly, the dry weight of the inoculated loose-leaf lettuce increased more than that of the control plants at 35, 43, 50, and 56 days after inoculation (Figure 4B). These data indicate that inoculation of the loose-leaf lettuce with strain 869T2 significantly enhanced plant growth. The weight increases of the inoculated loose-leaf lettuce plants were due to increases in average leaf width and length (Figure 4C,D), the number of leaves per plant (Figure 4E), total leaf area per plant and per leaf (Figure 4F,G), and plant height and width (Figure 4H,I). Furthermore, the root fresh weight of the inoculated loose-leaf lettuce plants increased 4.5- to 12.4-fold at 35, 43, 50, and 56 days after inoculation compared with the 0-day post-inoculation plants (Figure 4J); in contrast, that of the mock-inoculated control only increased 2.5- to 8.5-fold compared with the 0-day post-inoculation plants (Figure 4J). Additionally, the root dry weight and length increased more in the inoculated loose-leaf lettuce plants than in the control plants (Figure 4K,L). As seen in Figure 4M–O, overall plant size and leaf size increased after inoculation with strain 869T2, suggesting that strain 869T2 improves loose-leaf lettuce growth.

We also inoculated strain 869T2 into romaine lettuce and red leaf lettuce. The results shown in Figures S4 and S5 demonstrate that both kinds of lettuce grew taller and wider, had more and larger leaves, and had heavier aerial and belowground tissues after inoculation with strain 869T2 compared with the control plants. The chlorophyll contents of red leaf lettuce leaves were also higher in the 869T2-inoculated plants than the control plants (Figure S5H). These data collectively indicate that the three evaluated kinds of lettuce can grow significantly better after inoculation with strain 869T2.

**Figure 5:**
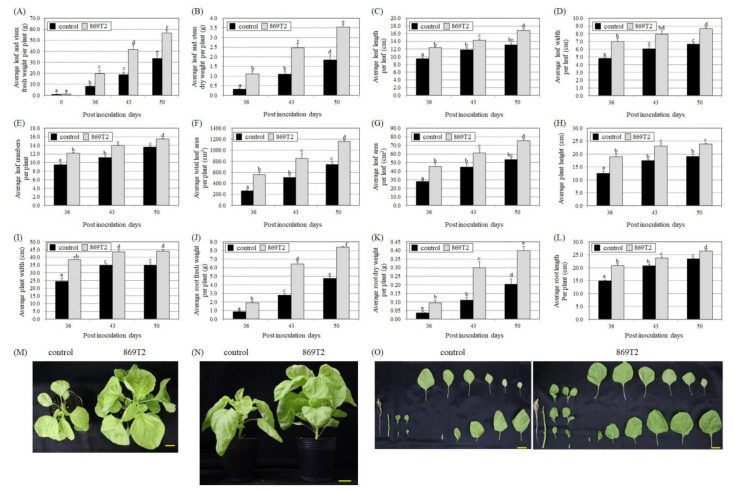
Inoculation of Chinese amaranth (*Amaranthus tricolor*) plants with *B. seminalis* strain 869T2 promoted growth of both aboveground and belowground parts after inoculation. At 36, 43, and 50 days after inoculation, the average values of leaf and stem fresh weight per plant (**A**), leaf and stem dry weight per plant (**B**), leaf length (**C**), leaf width (**D**), number of leaves (**E**), total leaf area per plant (**F**), leaf area per leaf (**G**), plant height (**H**), plant width (**I**), root fresh weight (**J**), root dry weight (**K**), and root length (**L**) of the control and inoculated plants were documented. Data are mean ± SE (standard error) from at least three independent bacteria inoculation experiments. More than 20 individual plants were examined for each bacteria inoculation assay. Data were analyzed using Duncan tests and means with different letters were significantly different (*p* < 0.05). Panels (**M**–**O**): photographs of the top-view (**M**), side-view (**N**), and the whole plant (**O**) for the control and 869T2-inoculated plants 43 days after inoculation. Yellow bar = 5 cm.

We also selected Chinese amaranth (*Amaranthus tricolor*) of the Amaranthaceae family to test the effect of strain 869T2 on its growth. At 36, 43, and 50 days after inoculation, the fresh weight of the 869T2-inoculated Chinese amaranth exhibited a 20.0- to 56.6-fold increase when compared to the 0-day post-inoculation plants, whereas the control plants only showed an 8.3- to 33.5-fold increase when compared to the 0-day post-inoculation plants (Figure 5A). Other plant growth parameters of the 869T2-inoculated and control plants were also examined 36, 43, and 50 days after inoculation (Figure 5). Figure 5 illustrates that the 869T2-inoculated Chinese amaranth individuals had more and larger leaves, were taller and wider, and had heavier and longer roots than the control plants. These data show that inoculating strain 869T2 into Chinese amaranth promoted its growth.

### 3.4. Effect of the B. seminalis Strain 869T2 on Flowering Time and Fruits of Hot Peppers and Okra

**Figure 6:**
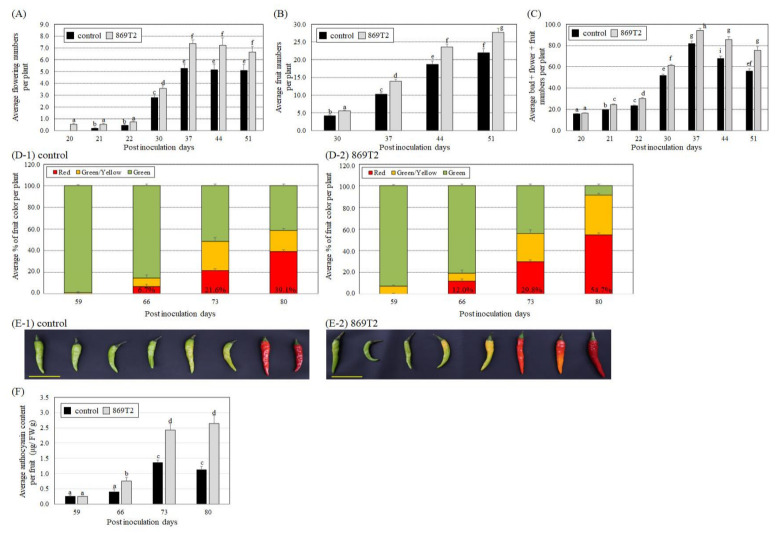
*B. seminalis* strain 869T2 inoculation caused hot pepper (*Capsicum annuum*) plants to have more flowers and fruits. The average values for the number of flowers (**A**), number of fruits (**B**), number of buds + flowers + fruits (**C**), and percentage of fruits of a given color (**D**) per plant were recorded for the control and inoculated plants after inoculation. Panel (**E**) shows photographs of fruits from the control (**E-1**) and inoculated (**E-2**) plants 73 days after inoculation. Yellow bar = 5 cm. After inoculation, the average anthocyanin content per fruit (**F**) was determined for the control and the 869T2-inoculated plants. Data are mean ± SE (standard error) from at least three independent bacteria inoculation experiments. More than 20 individual plants were examined for each bacteria inoculation assay. Duncan tests were used to analyze the data, and means with different letters were significantly different (*p* < 0.05).

Because *B. seminalis* strain 869T2 promoted the growth of several leafy vegetables, we next tested the effects of the strain 869T2 on the flowering and fruit production of hot pepper (*Capsicum annuum*) and okra (*Abelmoschus esculentus*). Hot pepper plants, from the Solanaceae family, were inoculated with strain 869T2 but we did not observe significant growth promotion effects on the aerial and root parts of the plants. However, we did observe that the 869T2-inoculated hot pepper plants flowered 20 days after inoculation; the number of flowers continually increased and had more than a 7-fold increase at 37 days after inoculation (Figure 6A). In the mock-inoculated control plants, we observed flowering 21 days after inoculation, and the number of flowers had only increased 5-fold at 37 days after inoculation (Figure 6A). The average number of fruits on the 869T2-inoculated plants was higher than that on the control plants at 30, 37, 44, and 51 days after inoculation (Figure 6B). The average numbers of flower buds, flowers, and fruits per plant were higher in the 869T2-inoculated plants than in the control plants beginning 21 days post-inoculation (Figure 6C). Furthermore, the percentages of hot pepper fruits with red and green/yellow coloring were higher in the 869T2-inoculated plants than in the control plants 59, 66, 73, and 80 days after inoculation (Figure 6D,E). Similarly, the average anthocyanin contents of the 869T2-inoculated plants were significantly higher than those of the control plants at 66, 73, and 80 days after inoculation (Figure 6F). However, the average length, width, and fresh weight of the fruits were not significantly different between the inoculated and control plants (Figure S6). Collectively, these data suggest that the inoculation of hot pepper with strain 869T2 could increase flowering and fruiting in hot pepper plants and accelerate fruit maturation.

**Figure 7:**
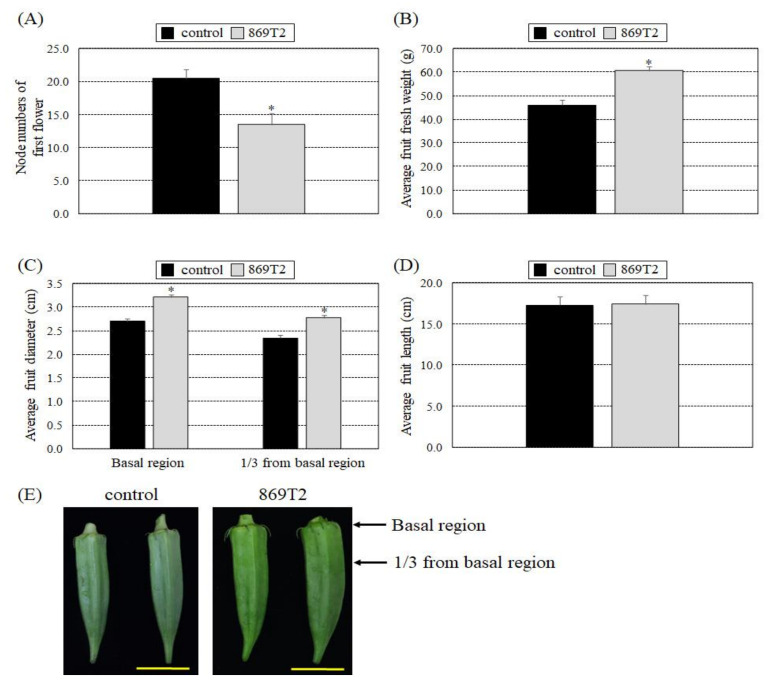
After inoculation with *B. seminalis* strain 869T2, okra (*Abelmoschus esculentus*) plants had heavier and wider fruits than control plants. The average values for the number of nodes of the first flower (**A**), fruit fresh weight (**B**), fruit diameter (**C**), and fruit length (**D**) were determined for the control and inoculated plants after inoculation. Data are mean ± SE (standard error) from at least three independent bacteria inoculation experiments. More than 20 individual plants were examined for each bacteria inoculation assay. * *p* < 0.05 compared with the control plants by pairwise Student *t*-tests. Panel (**E**) shows photographs of the fruits from the control and the inoculated okra plants. Yellow bar = 5 cm.

We subsequently examined the effects of strain 869T2 on okra, which belongs to the Malvaceae family. The overall plant size and weight were not significantly different between the 869T2-inoculated and control okra plants. We observed, however, that the number of nodes of the first flower was smaller in the 869T2-inoculated okra than in the control plants, suggesting that the 869T2-inoculated okra plants flowered earlier than the control plants (Figure 7A). In addition, the average fresh weight and diameter of the fruits from the 869T2-inoculated plants were greater than those of the control plants (Figure 7B,C), although the average fruit lengths were similar. These data demonstrate that the okra fruits became heavier and wider after inoculation with strain 869T2. In summary, inoculation of strain 869T2 into hot pepper and okra plants could cause plants to flower at earlier growth stages.

## 4. Discussion

The members of the genus *Burkholderia* belong to the class β-proteobacteria and have a broad distribution, residing universally in soil, water, and in association with plants, fungi, animals, and humans. Some *Burkholderia* species are plant pathogens in many vegetables and fruits, while others have been reported as opportunistic pathogens of humans and other animals [50]. However, many other *Burkholderia* species are beneficial to plants, suppressing plant diseases and promoting plant growth by various processes, including the production of antibiotics, secretion of allelochemicals, induction of pathogen resistance in plants, nitrogen fixation, or enhancing nutrient uptake by host plants [51,52]. These beneficial *Burkholderia* species are free-living or endophytic and form mutualistic associations with their host plants [52]. *Burkholderia* species’ high versatility and adaptability to different ecological niches rely on the high genomic plasticity of their large multichromosome genomes and the production of various bacteria secondary metabolites [50,53]. In this study, we characterized the endophytic bacterium *Burkholderia seminalis* strain 869T2 isolated from vetiver grass, which was recently described and included in the *Burkholderia cepacia* complex (Bcc). We have documented the IAA production, siderophore synthesis, and phosphate solubilization abilities of *B. seminalis* strain 869T2. Inoculations of strain 869T2 into tested plants demonstrated the plant growth promotion ability of this bacterium in several plant species from the Brassicaceae, Asteraceae, and Amaranthaceae families.

Plant endophytic bacteria can increase the nutrient uptake and biomass accumulation of host plants through the production or regulation of various plant hormones, such as auxin, cytokinin, gibberellins, and ethylene [1]. Indole acetic acid (IAA) is a naturally occurring auxin produced by several endophytic bacterial species through the L-tryptophan metabolism pathway. Tryptophan can exist in the exudates of plants and is utilized by the bacteria to synthesize auxin, which enhances the growth of host plants. Auxin is the major plant hormone that regulates various aspects of plant growth and development, such as root initiation and development, leaf formation, fruit development, floral initiation and patterning, phototropism, and embryogenesis [54]. Several plant-growth-promoting bacteria can synthesize IAA, including *Bacillus*, *Burkholderia*, and *Pseudomonas* species [1,34,35,45,46,47,51,55,56,57,58]. In this study, *Burkholderia seminalis* strain 869T2 was able to synthesize approximately 2.0 to 2.2 μg mL^−1^ IAA in the presence of tryptophan and increased both the aboveground and belowground biomass of tested plant tissues. Several previous reports also demonstrated that low levels of IAA stimulated primary root growth [1,2,5,8]. Similar to our observations, the *Burkholderia* sp. SSG that was isolated from boxwood leaves produced 2.9 to 4.5 μg mL^−1^ of IAA with tryptophan and had plant growth promotion ability in three boxwood varieties [55]. Additionally, *Burkholderia phytofirmans* strain PsJN, which was isolated from onion roots, showed higher IAA production, around 12 μg mL^−^^1^, with the addition of tryptophan and improved the growth of potato, tomato, maize, and grapevines [57,59]. Other *Burkholderia seminalis* strains can also synthesize IAA and have been reported to increase rice and tomato seedling growth [33,34]. These previous studies, along with our observations, suggest that *B. seminalis* strain 869T2 may be similar to other *Burkholderia* species and other plant-growth-promoting bacteria that utilize IAA to increase root growth, which may assist host plants in taking up nutrients from the surrounding environment and improve aerial tissue growth. Consistent with this hypothesis, we observed that plant size, height, fresh weight, dry weight, and total leaf areas of several tested plant species all significantly increased after inoculation with *B. seminalis* strain 869T2. It is known that the IAA can positively affect cell division, enlargement, tissue differentiation, root formation, and the control process of nutrition growth. The IAA can also function as a signal molecule to influence the expression of various genes involved in energy metabolism and other plant hormone synthesis, such as gibberellin and ethylene [1,2,5,54]. Interestingly, we observed earlier flowering in the 869T2-inoculated hot pepper and okra plants, suggesting that acceleration of plant growth rates might occur in these plants. In the future, transcriptome analysis of plant hormone response genes and energy-metabolic-related genes in the 869T2-inoculated plants might help us further decipher the possible mechanism of plant growth promotion ability of strain 869T2.

From the results of our study, we observed that *B. seminalis* strain 869T2 had a better IAA yield at a temperature range of 25 °C to 37 °C and pH of 6 to 9. Similarly, *Burkholderia pyrrocinia* strain JK-SH007 reached the maximum production of IAA at 37 °C and pH 7.0 [46]. Several other plant-growth-promoting bacteria, including *Bacillus siamensis*, *Bacillus megaterium*, *Bacillus subtilis*, and *Bacillus cereus*, had relatively higher IAA yields at temperatures of 2–135 °C and pH 7–8 [46,56]. Three different bacteria isolated from the rhizosphere of *Stevia rebaudiana* also exhibited greater production of IAA at a pH range of 6–9 and a temperature of 35 °C to 37 °C; these bacteria also increased the root and shoot biomasses of wheat and mung bean [45]. Various carbon sources are used as an energy source for IAA production and could enhance recycling of cofactors in bacterial cells [2,5,47,56,58]. Our results revealed that IAA yields of *B. seminalis* strain 869T2 were slightly better when glucose and fructose were used in media. Several previous publications also indicated that the ability of plant-growth-promoting bacteria to produce IAA was different, depending on the carbon source used in the media [5,45,46,47,56,58]. Results from these studies and our study demonstrated that IAA production by different plant-growth-promoting bacteria can be influenced by various factors, such as temperature, pH, carbon sources, culture conditions, and bacterial species. In this study, we utilized the colorimetric method to estimate the IAA amounts of *B. seminalis* strain 869T2 when grown in various in vitro conditions and media. Because the available tryptophan in the rhizosphere and root exudates of plants might be relatively lower than the tryptophan used in the media, the IAA production of *B. seminalis* strain 869T2 when grown in inoculated plants shall be determined with more sensitive and accurate methods, such as high-performance liquid chromatography (HPLC) or ultra-performance liquid chromatography (UPLC) systems.

Apart from the IAA production ability of *B. seminalis* strain 869T2, this bacterium exhibited siderophore production and phosphate solubilization activities. Iron is an important element for many biological processes in plant growth and development. Most iron in soils is present in the highly insoluble ferric (Fe^3+^) form, which is unavailable for plant absorption. Endophytic bacteria can yield iron-chelating agents such as siderophores, which bind ferric iron and help transport it into plant cells via root-mediated degradation of organic chelate, ligand exchange, or other mechanisms [1,8,59]. Phosphorus is another essential macronutrient for numerous metabolism processes in plants, such as biosynthesis of macromolecules, signal transduction, photosynthesis, and respiration. Most of the phosphorus in soil is insoluble and not available for root uptake to support plant growth. In order to increase the bioavailability of phosphorus for plants, certain endophytic bacteria turn insoluble phosphate into soluble forms via the processes of chelation, ion exchange, acidification, or production of organic acids [1,8,59]. Previous studies have also correlated siderophore production and phosphate solubilization abilities with the plant growth promotion traits of other *Burkholderia* species, such as the *Burkholderia* sp. SSG isolated from boxwood and the *Burkholderia* sp. MSSP isolated from root nodules of *Mimosa pudica* [55,60]. *Burkholderia cenocepacia* strain CR318, which was isolated from maize roots, significantly enhanced maize plant growth by solubilizing inorganic tricalcium phosphate [61]. Other studies have revealed that additional *Burkholderia* species also have the ability to solubilize inorganic phosphate to increase available phosphorous in agricultural soils and improve agricultural production [62,63,64]. In summary, both previous studies and our results suggest that the IAA synthesis, siderophore production, and phosphate solubilization abilities of *B. seminalis* strain 869T2 may collectively contribute to the growth enhancement observed in the several plant species tested here.

We successfully inoculated and reisolated *B. seminalis* strain 869T2, which was originally isolated from the monocot plant vetiver grass (*Chrysopogon zizanioides*) [18], in several eudicot plant species of the Brassicaceae, Asteraceae, Amaranthaceae, Solanaceae, and Malvaceae families. Strain 869T2 can significantly improve the growth of both the roots and aerial parts of *Arabidopsis* and several leafy vegetables, including ching chiang pak choi, pak choi, loose-leaf lettuce, romaine lettuce, red leaf lettuce, and Chinese amaranth. These results suggest that the endophytic bacterium strain 869T2 may have a wide host range. A similar observation was reported for *Burkholderia phytofirmans* strain PsJN, first isolated from onion roots [65], which enhanced the growth of *Arabidopsis*, switch-grass, potato, tomato, maize, wheat, and grapevines [57,59,66,67]. We did not observe significant growth improvement in hot pepper or okra plants after inoculation with strain 869T2; however, we did observe early flowering and better fruit development in these tested plants. These results suggest that the plant growth promotion abilities of strain 869T2 might be more apparent in crops with a shorter life cycle or that the latter two tested host plant species might not be fully compatible with this bacterium. The plant colonization process and growth promotion abilities of endophytic bacteria seem to be active processes that are regulated by different characteristics of both the host plants and bacteria [1,2,5,68]. In conclusion, our study revealed the potential of *Burkholderia seminalis* strain 869T2 for use as a bioinoculant in agriculture to improve plant growth and production.

## Data Availability

The data underlying this article are available in the article and in its online supplementary material.

## Supplementary Materials

The following are available online at https://www.mdpi.com/article/10.3390/microorganisms9081703/s1, Figure S1: *B. seminalis* strain 869T2 exhibited siderophore production and phosphate solubilization abilities that were confirmed using plate assays, Figure S2: the *Arabidopsis thaliana* (ecotypes BL-1, UE-1, and Dijon-G) exhibited better growth after inoculation with *B. seminalis* strain 869T2 grown at 30°C, Figure S3: the pak choi (*Brassica rapa* L. R. Chinensis Group) grew better after inoculation with *B. seminalis* strain 869T2, Figure S4: the romaine lettuce (*Lactuca sativa* L. var. *romana*) plants became bigger and heavier after inoculation with *B. seminalis* strain 869T2, Figure S5: the *B. seminalis* strain 869T2 inoculation increased the growth of red leaf lettuce (*Lactuca sativa* L. var. *crispa*) plants 44 and 77 days after inoculation, Figure S6: the *B. seminalis* strain 869T2 inoculation did not significantly affect the fruit size or fresh weight of the hot pepper (*Capsicum annuum*) plants.
