# Supplementary material for: A Plant Endophytic Bacterium, Burkholderia seminalis Strain 869T2, Promotes Plant Growth in Arabidopsis, Pak Choi, Chinese Amaranth, Lettuces, and Other Vegetables
Source: Microorganisms. 2021 Aug 10;9(8):1703. doi: 10.3390/microorganisms9081703 (PMC8401003; doi:10.3390/microorganisms9081703)
Supplement: Supplementary file 1 [file microorganisms-09-01703-s001.zip › microorganisms-1326710-supplementary.pdf]

Supplementary Materials

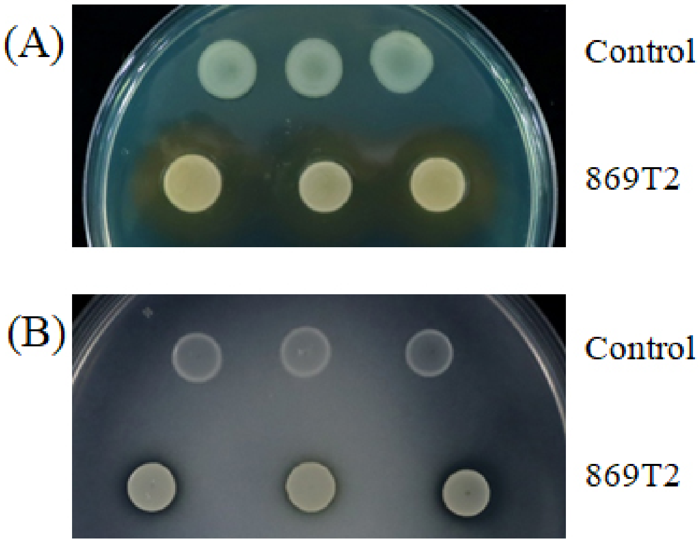

Supplementary Figure S1. *B. seminalis* strain 869T2 exhibited siderophore production and phosphate solubilization abilities that were confirmed using plate assays. Panel A: the strain 869T2 colonies turned yellow colors while the negative control *Escherichia coli* (*E. coli*) strain remained blue colors. Panel B: the strain 869T2 had a clear halo zone around the colony while the negative control *E. coli* strain had no such halo.

.

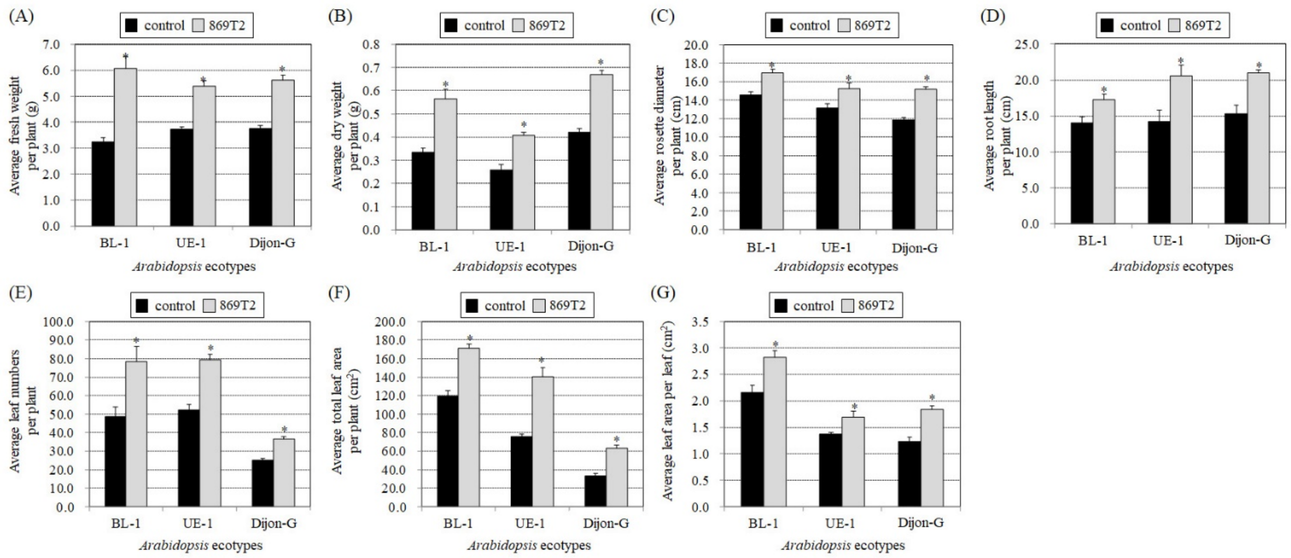

Supplementary Figure S2. The *Arabidopsis thaliana* (ecotypes BL-1, UE-1, and Dijon-G) exhibited better growth after inoculation with *B. seminalis* strain 869T2 grown at 30°C. After inoculation, the average values for fresh weight (Panel A), dry weight (Panel B), rosette diameter (Panel C), root length (Panel D), number of leaves (Panel E), total leaf area per plant (Panel F), and leaf area per leaf (Panel G) were determined for the control and the inoculated plants. Data are mean±SE (standard error) from at least three independent bacteria inoculation experiments. More than 20 individual plants were examined for each bacteria inoculation assay. * $p < 0.05$ compared with the control plants by pairwise Student t tests.

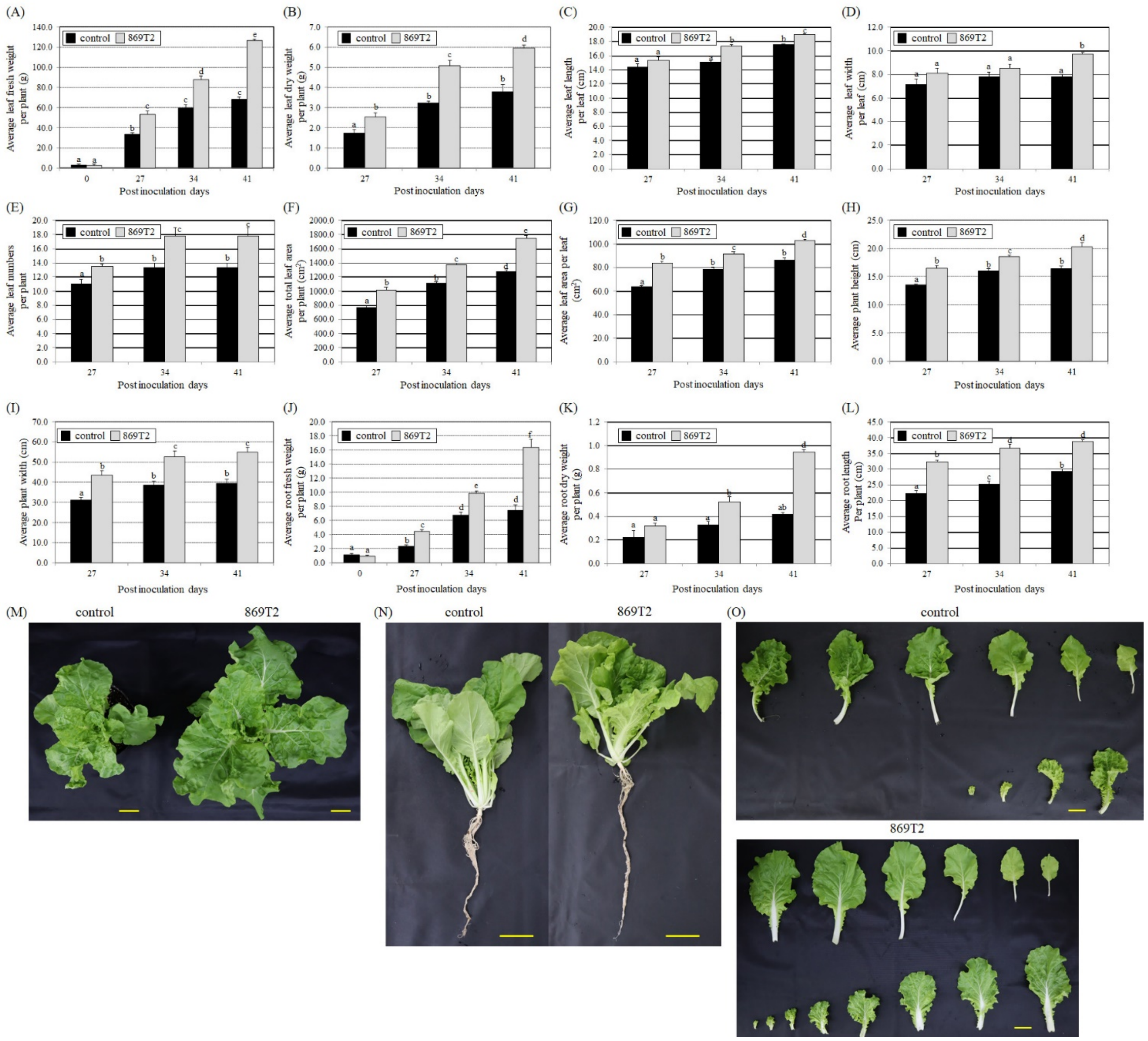

Supplementary Figure S3. The pak choi (*Brassica rapa* L. R. Chinensis Group) grew better after inoculation with *B. seminalis* strain 869T2. At 27, 34, and 41 days after inoculation, the average values for leaf fresh weight (Panel A), leaf dry weight (Panel B), leaf length (Panel C), leaf width (Panel D), number of leaves (Panel E), total leaf area per plant (Panel F), leaf area per leaf (Panel G), plant height (Panel H), plant width (Panel I), root fresh weight (Panel J), root dry weight (Panel K), and root length (Panel L) were determined for the control and the inoculated plants. Data are mean±SE (standard error) from at least three independent bacteria inoculation experiments. More than 20 individual plants were examined for each bacteria inoculation assay. Duncan tests were used for statistical analysis, and means with different letters were significantly different ($p < 0.05$). Panels M-O: photographs of the top view (M), side view (N), and total leaves (O) of the mock-inoculated control and the 869T2-inoculated plants 27 days after inoculation. Yellow bar = 5 cm.

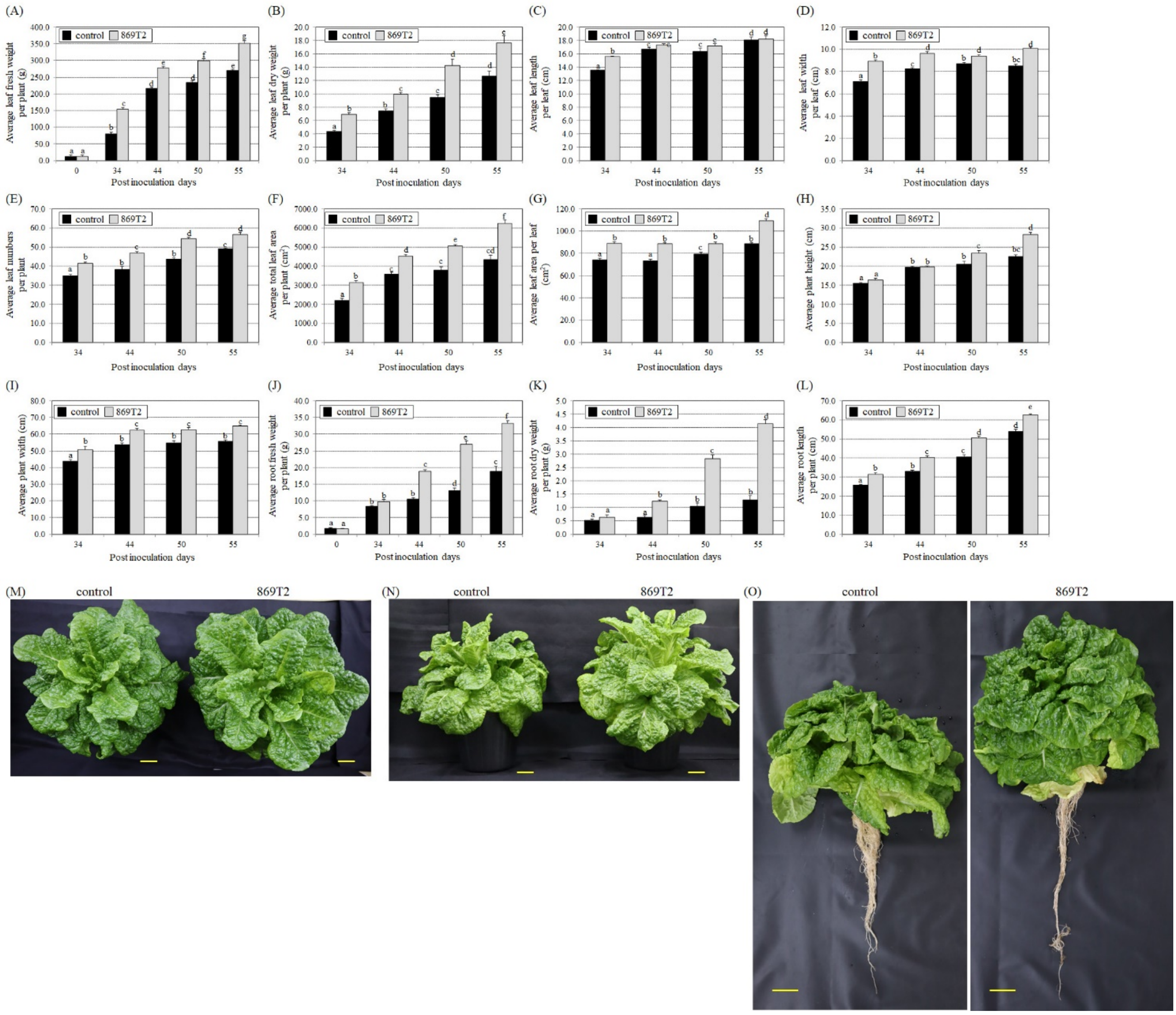

Supplementary Figure S4. The romaine lettuce (*Lactuca sativa* L. var. *romana*) plants became bigger and heavier after inoculation with *B. seminalis* strain 869T2. At 34, 44, 50, and 55 days after inoculation, the average values for leaf fresh weight (Panel A), and leaf dry weight (Panel B) per plant, leaf length (Panel C), leaf width (Panel D), number of leaves (Panel E), total leaf area per plant (Panel F), leaf area per leaf (Panel G), plant height (Panel H), plant width (Panel I), root fresh weight (Panel J), root dry weight (Panel K), and root length (Panel L) were examined for the control and the inoculated plants. Data are mean±SE (standard error) from at least three independent bacteria inoculation experiments. More than 20 individual plants were examined for each bacteria inoculation assay. Data were analyzed using Duncan tests, and means with different letters were significantly different ($p < 0.05$). Panels M-O: photographs of the top view (M), side view (N), and the whole plant (O) for the control and inoculated plants 50 days after inoculation. Yellow bar = 5 cm.

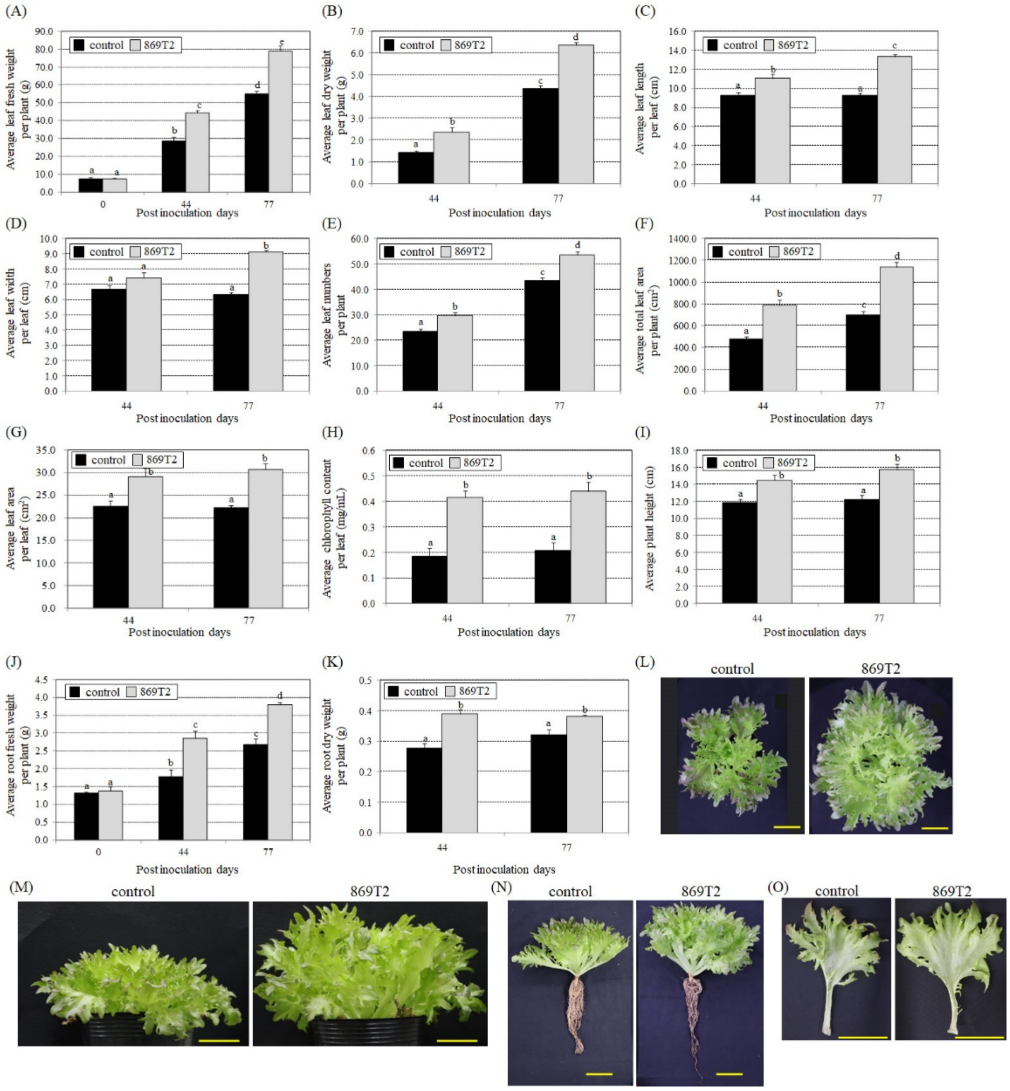

Supplementary Figure S5. The *B. seminalis* strain 869T2 inoculation increased the growth of red leaf lettuce (*Lactuca sativa* L. var. *crispa*) plants 44 and 77 days after inoculation. The average values of the leaf fresh weight (Panel A) and leaf dry weight (Panel B) per plant, leaf length (Panel C), leaf width (Panel D), number of leaves (Panel E), total leaf area per plant (Panel F), leaf area per leaf (Panel G), chlorophyll content per leaf (Panel H), plant height (Panel I), root fresh weight (Panel J), and root dry weight (Panel K) were determined for the control and inoculated plants after inoculation. Data are mean±SE (standard error) from at least three independent bacteria inoculation experiments. More than 20 individual plants were examined for each bacteria inoculation assay. Data were analyzed using Duncan tests, and means with different letters were significantly different ($p < 0.05$). Panels L-O: photographs of the top view (L), side view (M), whole plant (N), and a single leaf (O) for the control and the inoculated plants 44 days after inoculation. Yellow bar = 5 cm.

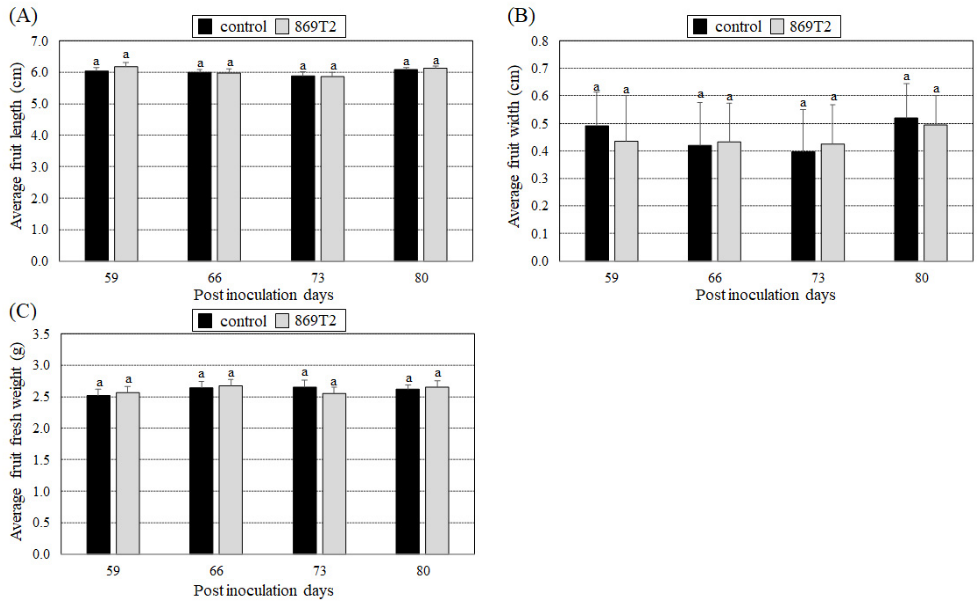

Supplementary Figure S6. The *B. seminalis* strain 869T2 inoculation did not significantly affect the fruit size or fresh weight of the hot pepper (*Capsicum annuum*) plants. At 59, 66, 73, and 80 days after inoculation, the average fruit length (Panel A), fruit width (Panel B), and fruit fresh weight (Panel C) of the control and the inoculated plants were documented. Data are mean±SE (standard error) from at least three independent bacteria inoculation experiments. More than 20 individual plants were examined for each bacteria inoculation assay. Duncan tests were used to analyze the data, and means with the same letters were not significantly different ($p < 0.05$).
